# Vitamin A status and body pool size of infants before and after consuming fortified home-based complementary foods

**DOI:** 10.1186/s13690-016-0121-4

**Published:** 2016-03-07

**Authors:** Sam Newton, Seth Owusu-Agyei, Kwaku Poku Asante, Esi Amoaful, Emmanuel Mahama, Samuel Kofi Tchum, Martha Ali, Kwame Adjei, Christopher R. Davis, Sherry A. Tanumihardjo

**Affiliations:** 1 Kintampo Health Research Centre, P. O. Box 200, Kintampo, Brong Ahafo Region Ghana; 2 Nutrition Department, Ghana Health Service, Accra, Ghana; 3 Department of Nutritional Sciences, University of Wisconsin-Madison, Madison, WI USA

**Keywords:** Body pool size, Complementary foods, Home fortification, Human infants, MRDR, Vitamin A, ^13^C-RID

## Abstract

**Background:**

Home fortification using sachets of micronutrient powder (e.g. “Sprinkles”) is a food-based approach offering an alternative to high dose vitamin A (VA) supplements for infants. The primary objective was to investigate the impact of VA-home fortification on infant VA pool size. The secondary objective was to compare VA status of infants assessed by the modified relative dose response (MRDR) test before and the ^13^C-retinol isotope dilution (^13^C-RID) test in the same infants after vitamin A supplementation.

**Methods:**

A randomized-controlled trial was conducted in 7–9 month old infants in Ghana. Eligible children were randomly allocated to receive a daily sachet of “Sprinkles” with or without VA for 5 months added to complementary foods. The MRDR test indirectly determined VA liver reserves at baseline and the ^13^C-RID determined VA body pool at follow-up in the same cohort of children.

**Results:**

At baseline, the MRDR values (95 % CI) for infants were comparable in the intervention and control groups: normal at 0·032 (SD 0·018) (0·025–0·038) and 0·031 (SD 0·018) (0·024–0·038), respectively. After intervention, total body stores (TBS) and liver retinol concentrations did not differ between intervention and control groups; TBS were 436 (SD 303) and 434 (SD 186) μmol, respectively, and estimated liver concentrations were 0·82 (SD 0·53) and 0·79 (SD 0·36) μmol/g liver, indicating adequate reserves in all children.

**Conclusions:**

Both the MRDR and ^3^C-RID tests confirmed that the infants had adequate VA status before and after home fortification of their complementary foods. These tests offered more information than serum retinol concentrations alone, which predicted VA deficiency using current suggested cutoffs not corrected for inflammation status.

## Background

The growth rate of breast-fed infants in developing countries during the first 6 months of life is comparable to that of infants in developed countries. However, infants in developing countries deviate from this satisfactory growth pattern after this period [1]. This has been attributed to lack of nutrient-dense complementary foods and is further exacerbated by persistent micronutrient deficiencies [2] thus making children in developing countries vulnerable to diseases and death during the weaning period. One means of addressing this problem in poor communities where infants and young children consume monotonous cereal-based diets, is by feeding infants complementary foods containing micronutrients, such as vitamin A (VA), iron and zinc sprinkled on the food immediately before feeding [3–5].

Vitamin A deficiency is a public health problem in many countries and diminishes the ability of young infants to fight infections predisposing them to an increased risk of early death [6]. Infections occurring during the infant’s life lead to increased risk of morbidity [7, 8], VA excretion in urine, and increased VA requirements [9]. High dose supplements are an effective way to ward off the deleterious effects of VA deficiency [10], and reduce mortality [11] and severe morbidity [12, 13] in children 1–5 y of age in less developed countries. Children are born with low VA stores and depend on their mother’s milk for VA. Before an infant is introduced to complementary foods, the mother may not be able to provide enough VA to boost the child’s liver stores if she herself has low VA stores [14] or does not consume rich sources of VA during lactation.

Home fortification is a food-based approach offering an alternative to administration of high dose VA supplements directly to infants and young children [3]. A novel practical formulation of micronutrient powders in single dose sachets, commonly called “Sprinkles”, was developed for home fortification of weaning foods to address the problem of micronutrient deficiency in young infants. “Sprinkles” can be added once daily to any complementary food immediately before serving. Sachets typically contain iron and zinc; vitamins A, C, and D; and folic acid [15]. Sprinkles can be used to meet the infants’ high VA requirements for rapid growth after 6 months of age [15–17].

The success of home-based strategies needs to be evaluated by assessing VA status [18]. Serum retinol concentrations, which are homeostatically controlled yet depressed during times of infection due to the acute phase response [19, 20], are only useful when liver reserves are severely depleted but many children suffer from a marginal VA status [21]. The modified relative dose response (MRDR) test indirectly determines VA liver reserves. As liver VA reserves become depleted, *apo*-retinol-binding protein accumulates in the liver. A challenge dose of 3,4-didehydroretinyl acetate is administered and the response of 3,4-didehydroretinol (DR)-*holo*-retinol-binding protein complex is measured in the serum ~5 h after dosing [21–23]. The MRDR test is a categorical indicator of VA status and is typically positive at <0.1 μmol retinol/g liver [21]. The MRDR test distinguishes between moderately inadequate and adequate VA status, based on the ratio of DR to retinol (DR:R) in serum after dosing [24]. Stable isotopes are used to determine the VA body pool by using deuterium or ^13^C-retinol as a tracer [25, 26]. The tracer dilution technique is the only indirect measure that provides a quantitative estimate of total body VA pool size [27] and stable isotopes lack the potential deleterious effects of radioisotopes on human health [28].

The primary objective of this study was to investigate the impact of VA-home fortification on infant VA pool size using the ^13^C-retinol isotope dilution (^13^C-RID) test at follow-up among children who received “Sprinkles” with or without VA added to complementary foods. VA status of infants was determined at baseline with the MRDR test because it requires a smaller volume of blood and is less expensive to analyse than the ^13^C-RID test. Thus, although the two methods were not used concurrently at baseline and endline, the secondary objective was to use the MRDR test and the ^13^C-RID test in the same cohort of infants because this has not been done before.

## Methods

### Study site

The trial was carried out in 7 villages surrounding Kintampo located in the Brong Ahafo Region of Ghana. The district has a resident population of about 140,000, the majority of whom have a relatively poor socioeconomic status [29]. Anthropometric data also indicated a prevalence of stunting of 32 % and wasting of 4 % among children aged 12 months [30].

### Participants and study interventions

This community-based study included infants aged 7–9 months (*n* 93) and their ages were verified by inspecting their vaccination cards. Children of this age were selected to ensure that weaning had been established after they were identified by trained field workers. Eligible children were enrolled at home and randomly allocated to receive daily “Sprinkles” with or without VA using a computer generated random number table. Eligibility criteria included willingness of mothers to provide consent, to stay in the study area throughout the study duration and to feed the child with the contents of the micronutrient sachets. The child was also expected to eat complementary foods in addition to breast milk, and haemoglobin needed to be >70 g/l. Those in the VA group received a daily dose of a powdered fortificant (MNP-Sprinkles; Mumbai, India) containing 12 · 5 mg of elemental iron (as microencapsulated ferrous fumarate) plus ascorbic acid (30 mg), retinyl palmitate (400 μg RAE), and zinc (5 mg). The control group received a similar fortificant that did not contain VA. Blinding of the intervention was carried out by a neutral group of persons who packaged the supplements in identical packages with codes unknown to the investigators. Field workers delivered weekly supplements to mothers for use 7 d/week. At the conduct of this trial, VA supplementation was a national policy given to infants when they reached 6 months of age through national campaigns. Children enrolled in this study were excluded from taking the routine VA supplementation until the end of the 5-month study by marking their vaccination cards stating that they were enrolled in another study and should not be given routine VA supplementation at 6 months of age as is the practice in Ghana. This was effectively ensured by inspecting the identity cards of all children enrolled to determine if they were part of any other ongoing studies or programs that routinely administer VA supplementation. Infants were followed for 5 months. Mothers were instructed to mix a single sachet of “Sprinkles” with a small amount of food and to add water and sugar as needed to ensure that the child consumed the entire sachet. Breast feeding is universal in this area and mothers were not prevented from breast feeding their infants during the intervention. Where there were two eligible children in a household, only one was randomly selected. This was done to prevent contamination if it happened that the two children belonged to different groups and they happened to share food with each other.

### Sample size

Sample size was based on previous stable isotope work done by Tondeur et al. [31] in Kintampo. We estimated that 15 infants per group would be sufficient to detect a 5 % difference in VA pool size with a 5 % SD on the basis of a type 1 error set at 0·05 and a 0·8 probability of detecting a true difference between the two groups. Incidentally, at the time this study was performed, fifteen children per group was considered adequate for the determination of the VA body pool size for supplements as stated by the Vitamin A Tracer Task Force [27]. The primary objective served as the basis for the sample size calculation but the sample size requirements for the MRDR test to be descriptive of the VA status as stated by the Vitamin A Tracer Task Force [27] was larger and hence the need for more children to be recruited.

### Study procedures

A detailed explanation of the purpose, risks, and benefits were verbally explained and consent was sought for children’s participation from their mothers. In the presence of a witness, mothers who were literate signed a consent form but fingerprints were obtained for those who could not sign. The child of each consenting mother was issued a study ID card containing identification information, which was used by trained staff to replenish their weekly supply of Sprinkles and for database management. Information on compliance was obtained by collecting the used empty sachets weekly from mothers and because the study was double-blind, compliance was expected to be similar in both groups; however, the allocation of the empty sachets were not verified to determine which group they belonged to in order not to disclose the groups to which the children had been assigned.

All information collected was considered confidential and was de-identified. The Institutional Ethics Committee of the Kintampo Health Research Centre (Office for Human Research Protections Federal Wide Assurance Number 00011103 and IRB registration number 0004854) approved the study protocol. The study was registered with clinical trials.gov NCT 01751009.

Information was collected on socioeconomic status (occupation), marital, and education status. Morbidity questionnaires were used to collect health data by fieldworkers for the 5 months through surveillance fortnightly, the health of the child was assessed and information was collected on whether the child had been taken to a health facility within the past two weeks. At the beginning and end of the 5 months study period, anthropometric, haemoglobin [32], CRP [33] (QuickRead, Orion Diagnostica, Finland), and ferritin (Spectro Ferritin, Ramco Laboratories USA) [34, 35] assessments were carried out. Haemoglobin concentrations were measured with the use of a portable HEMOCUE-Hemoglobin photometer (Hemocue Inc, Angelholm, Sweden). Haemoglobin was considered low if <100 g/l [36]. Depleted iron stores were defined as ferritin < 12 μg/l and elevated CRP was defined as >5 mg/L.

### Modified relative dose response test

The MRDR test involved giving an oral dose of 5 · 3 μmol 3,4-didehydroretinyl acetate dissolved in 290 μl corn oil in the morning using a 0 · 3 ml insulin syringe. The children were dosed at their homes and 5 h later a heel prick blood sample (~500 μl) was taken. The samples were stored on ice away from light in a cooler until transported to the laboratory. Clotted blood samples were centrifuged at 600 X *g* for 10 min and the serum was stored at –20 °C until shipped. After completion of the trial, samples were shipped frozen to the Vitamin A Assessment Laboratory at University of Wisconsin-Madison. All samples arrived frozen and were immediately stored at -80 °C until analysis. The samples were analysed for DR and R using a standardised method developed specifically for small serum volumes [37]. MRDR values (DR:R) >0 · 06 were used to indicate VA deficiency.

### Extraction and high-pressure liquid chromatography (HPLC) procedures

The standard HPLC method was followed as published for 200 μl serum [37] except three extractions were made with 300 μl hexanes instead of two [38].

### Description of ^13^C-retinol isotope dilution test

Blood samples of 7 children from both groups were randomly taken in May 2010 after the last sachets were used to serve as a measure of natural abundance of ^13^C [39]. The remaining [33] children (14 VA group; 19 control) were given an oral 1 μmol (288 μg retinol equivalents) dose of ^13^C_2_-retinyl acetate followed by 14 d to allow for tracer mixing with the retinol pool in children [40, 41]. Blood (2 ml) was collected from 33 infants for assessment of the VA liver stores and results were obtained for 24 infants because insufficient serum was obtained from some infants. The optimal amount of serum required for the test is 1 · 5 ml, although we were able to get reliable readings on 0 · 5 ml. The samples were analysed using the method of Howe et al. [39] modified by Valentine et al. [42]. The gas chromatography/combustion/isotope ratio mass spectrometer was run as previously described by Howe et al. [39].

### Calculation of total body vitamin A stores

Total body VA was calculated using the following mass balance equation, substituting for c, and rearrangement:

$$ \begin{array}{c}\hfill \left({\mathrm{F}}_{\mathrm{a}}\mathrm{x}\ \mathrm{a}\right) + \left({\mathrm{F}}_{\mathrm{b}}\mathrm{x}\ \mathrm{b}\right) = \left({\mathrm{F}}_{\mathrm{c}}\mathrm{x}\ \mathrm{c}\right)\hfill \\ {}\hfill \mathrm{c} = \mathrm{a} + \mathrm{b}\hfill \\ {}\hfill \mathrm{b} = \mathrm{a}\ \left({\mathrm{F}}_{\mathrm{a}} - {\mathrm{F}}_{\mathrm{c}}\right)\ /\ \left({\mathrm{F}}_{\mathrm{b}} - {\mathrm{F}}_{\mathrm{c}}\right)\hfill \end{array} $$

Where F_a_ = atom percent (%At) of the dose*0 · 01 = 0 · 1 (2 of 20 atoms labeled), F_b_ = %At at baseline*0 · 01 based on the mean of natural enrichment samples, and F_c_ = %At at day 14 after dosing*0 · 01 (%At for each of the individual results). Additionally, a = μmol VA absorbed from the dose, which is assumed to be 80 % in this group of infants who are susceptible to multiple infections [41, 43], b = uncorrected body pool at baseline (unknown), and c = μmol VA in body pool after dosing = a + b. It is then corrected for loss of tracer in the body over the 14 d by accounting for the half-life of retinol in young children [44], so the corrected body pool of VA = b x e^(-kt) where k = ln(2)/32 and t = time in days of the serum collection after the dose was administered. Finally, the total body stores (TBS) were corrected for the serum to liver ratio of 0 · 8 because the infants were not fed a low-VA containing diet during the equilibration period, which is supported by one human study [45]. Total liver reserves were assumed to be 80 % of TBS and liver weight was assumed to be 4 % of total body weight in these infants [43, 46]. Of the 14 completing children in the intervention group who were actually tested using stable isotopes, 10 were males and 4 were females, and for the 19 in the control group, 7 were males and 12 were females.

### Data management and statistical analyses

Field supervisors checked all forms manually for completeness. Forms were double entered on computers, range and consistency checks performed, and discrepancies resolved with reference to the original form using Microsoft Visual Foxpro version 9 · 0 Data Management Software. Data were analysed using Stata version 11. Simple descriptive analysis of baseline measures (e.g. demographic, socioeconomic, biochemical) was performed across the treatment groups to confirm their comparability. Categorical demographic characteristics were summarised as proportions, while continuous variables were summarised as means. Differences in means of quantitative variables, such as ferritin, MRDR, and CRP, between intervention and controls at baseline and endline were evaluated using t-tests. Normality of residuals for isotopic data was assessed by the Shapiro-Wilk test. Non-parametric analysis was carried out on ranked data. Anthropometric indices of height-for-age (HA), weight-for-age (WA), and weight-for-height (WH) were expressed as z-scores using the WHO Anthro for personal computers, Version 3 · 1, 2010. *P* < 0 · 05 was considered statistically significant.

## Results

### Enrolment of subjects

**Fig. 1:**
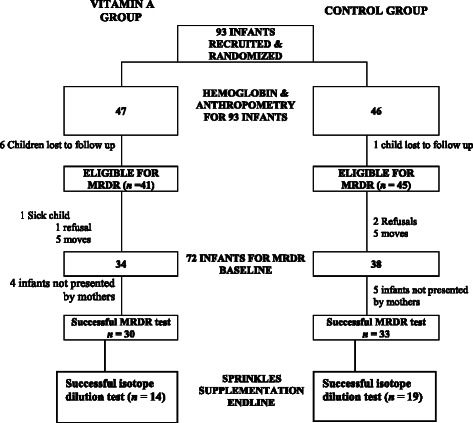
Numbers of infants at each stage in the trial, and the reasons for any losses to follow up

The studied children were enrolled from January through June 2010. There were 30/47 (63 · 9 %) males in the intervention group and 19/46 (41 · 3 %) in the control group. Characteristics of mothers of children in both groups were similar (Table 1). Seven infants were lost to follow-up before blood samples could be collected for VA analyses leaving 86 eligible children (Fig. 1). Ten mothers moved out of the study area during the farming season, a mother reported that her child was sick and three mothers refused to allow blood samples of their infants to be collected. Blood samples were collected from a total of 72 children for haemoglobin analyses.

**Table 1:** Comparison of age of children and baseline data of mothers in the intervention and control groups^a^

	Intervention		Control		*P*
	*n* 47		*n* 46		
	Number	Percent	Number	Percent	
Parity					
1–3	26	55 · 3	26	56 · 5	
4–6	14	29 · 8	12	26 · 0	0 · 09
>6	7	14 · 9	8	17 · 4	
Education					
None	29	61 · 7	31	67 · 4	
Primary	7	14 · 9	5	10 · 9	0 · 80
Middle	8	17 · 0	7	15 · 2	
Secondary	3	6.4	2	4 · 3	
Unknown	0		1	2 · 1	
Marital status					
Married	32	68 · 1	29	63 · 0	
Living together	12	25 · 5	12	26 · 0	0 · 71
Widowed	0	0	1	2 · 2	
Divorced	0	0	1	2 · 2	
Single unmarried	3	6 · 4	3	6 · 5	
Occupation					
Farmer	38 · 0	80 · 8	34 · 0	73 · 9	
Trader	5 · 0	10 · 6	5 · 0	10 · 9	0 · 59
Professional	4 · 0	8 · 5	7 · 0	15 · 2	
Age, months	Mean	Range	Mean	Range	
Baseline	8 · 2	7 · 9–8 · 1	8 · 4	8 · 1–8 · 7	0 · 30
Endline	14 · 1	13 · 7–14 · 1	14 · 4	14 · 0–14 · 7	0 · 23

^a^No significant difference was noted among treatment groups for all characteristics shown

### Vitamin A status in infants

Out of 93 children enrolled, 63 blood samples were finally collected for MRDR testing at baseline to determine VA status. Even though 72 children were eligible, nine infants were not presented by their mothers for the final blood draw for the MRDR test due to suspected concerns with blood draw. The MRDR test (*n* 30 and 33 in the intervention and control groups, respectively) showed that there was no difference in the VA status between the groups even when infants with high CRP were excluded (Table 2). At baseline, the mean ratio (95 % CI) of MRDR for infants in the intervention group represented sufficient vitamin A status 0 · 032 (0 · 025–0 · 038) (SD 0 · 018), values did not differ from those of the control group, i.e., 0 · 031 (0 · 024–0 · 038) (SD 0 · 018). In contrast, the mean serum retinol concentrations were 0 · 812 (SD 0 · 238) (95 % CI 0.73–0.90) and 0 · 781 (SD 0 · 266) (95 % CI 0.69–0.87) μmol/L for the intervention and control groups, respectively, with 34.9 % of the children having a serum retinol concentration below 0 · 7 μmol/L, which is used as a cutoff value for VA deficiency. The MRDR and serum retinol concentrations were not correlated (*r* = 0.167, *P* = 0.19). After 5 months supplementation, the vitamin A status was assessed as endline on a subgroup of infants in both groups using the ^13^C-RID test. Liver retinol concentrations did not differ between groups (*P* = 0 · 87) and all children had adequate status. The intervention and control groups had TBS of 436 (SD 303) and 434 (SD 186) μmol, respectively. The estimated liver reserves were 0·82 (SD 0·53) and 0·79 (SD 0·36) μmol/g of liver for the intervention and control groups, respectively. Even removing the results of TBS of three potential outliers (545 – 697 μmol) out of the total 24 infants did not demonstrate a significant difference in the liver stores and therefore they were left in the statistical analysis. In this group of infants, both the MRDR and isotope dilution tests indicated adequate liver reserves in all children.

**Table 2:** Infant vitamin A status, hematologic and anaemia indexes at baseline and end line in intervention and control groups

	Intervention		Control		
Low vitamin A status^y^	Number	%	Number	%	
All infants	2/29	6 · 9	2/34	5 · 9	0 · 87
Excluding infants with high CRP^d^	2/22	9 · 1	2/26	7 · 7	0 · 86
Adequate vitamin A by MRDR^χ^					
All infants^χ^	27/29	93 · 1	32/34	88 · 2	0 · 86
Excluding infants with high CRP^d^	20/22	90 · 9	24/26	92 · 3	0 · 86
Baseline indexes	Mean		Mean		
Haemoglobin (g/l)	103		101		
	Number	%	Number	%	
Prevalence of anaemia^b^	18/47	38 · 3	20/46	42 · 5	0 · 61
Prevalence of low ferritin^c^	0/32	0.0	1/34	2 · 9	0 · 33
Prevalence of elevated CRP^d^	7/32	21 · 9	8/34	23 · 5	0 · 87
Endline indexes	Mean		Mean		
Haemoglobin (g/l)	111		108		
	Number	%	Number	%	
Prevalence of anaemia^b^	8/34	23 · 5	12/38	31 · 6	0 · 44
Prevalence of low ferritin^c^	0/16	0 · 0	0/17	0.0	-
Prevalence of elevated CRP^d^	8/17	47 · 0	7/17	41 · 2	0 · 73

^a^χ ± SD

^b^Defined as haemoglobin concentration <10 g/dl

^c^Depleted iron stores defined as ferritin < 12 μg/l

^d^Elevated CRP > 5 mg/L

^y^low vitamin A status MRDR ≥ 0 · 06

^χ^Adequate vitamin A status MRDR < 0 · 06

### Anthropometry and haematological indices

Weight and height of enrolled children did not differ at baseline and endline. There was no change observed in other anthropometric indices between the intervention and the control groups Table 3. The two groups were not significantly different with respect to their anaemia and CRP status at baseline. At the end of the supplementation period, more infants tended to be anaemic in the control group as compared with the intervention group (23 · 5 % vs. 31 · 6 %) but this was not significant (Table 2).

**Table 3:** Infant anthropometric status (z-scores) at baseline and end line in intervention and control groups

	Intervention			Control			*p*
Baseline anthropometry	Mean	SD	Range	Mean	SD	Range	
Child weight (kg)	7 · 6	1.03	7 · 20, 7 · 90	7 · 3	1.04	7 · 0, 7 · 60	0 · 19
Child height (cm)	68 · 2	3.19	67.3, 69.2	68 · 4	3.01	67 · 5, 69 · 3	0 · 79
Height-for-age^a^	-0 · 8	1 · 16	-1 · 18, -0 · 50	-0 · 7	1 · 28	-1 · 09, -0 · 33	0 · 62
Weight-for-age^a^	-1 · 0	1 · 06	-1 · 32, -0 · 69	-1 · 2	1 · 27	-1 · 60, -0 · 85	0 · 37
Weight-for-height^a^	-0 · 6	1 · 07	-0 · 95, -0 · 32	-1 · 1	1 · 12	-1 · 40, -0 · 72	0 · 06
Endline anthropometry							
Child weight (kg)	8 · 3	1.77	7 · 70, 8 · 90	8 · 3	1.74	7 · 70, 8 · 90	0 · 95
Child height (cm)	74.1	3.30	72 · 9, 75 · 3	74 · 5	3.31	73 · 4, 75 · 6	0 · 61
Height-for-age^a^	-1 · 4	1 · 17	-1 · 79, -0 · 95	-1.2	1 · 16	-1 · 55, -0 · 77	0 · 46
Weight-for-age^a^	-1 · 3	0 · 89	-1 · 64, -1 · 00	-1.3	1 · 16	-1.67, -0.90	0 · 9
Weight-for-height^a^	-0 · 9	0 · 72	-1 · 19, -0 · 67	-1.0	1 · 21	-1.43, -0.61	0 · 7

^a^χ ± SD

### Morbidity assessment for infants during biweekly visits

An assessment of clinical conditions was carried out every other week from the start of supplementation until the end of the 5 months period. There were no differences in any of the conditions assessed, but more infants sought treatment in the intervention group compared with the control group during weeks 12 and 14 (Table 4).

**Table 4:** Comparison of morbidity reports for infants at 2 week visits between intervention and control groups^a^

	Intervention		Control		*P*	Intervention		Control		*P*
	Number	Percent	Number	Percent		Number	Percent	Number	Percent	
	2 weeks		2 weeks			4 & 6 weeks		4 & 6 weeks		
Cough	8/40	20 · 0	6/38	15 · 8	0 · 63	11/82	13 · 4	7/76	9 · 2	0 · 40
Diarrhoea	12/40	30 · 0	11/38	28 · 9	0 · 92	19/82	23 · 2	24/76	31 · 6	0 · 23
Refuse breast	2/40	5 · 0	0/38	0 · 0	0 · 16	4/82	4 · 9	2/76	2 · 6	0 · 46
Feverishness	5/40	12 · 5	4/38	10 · 5	0 · 78	13/82	15 · 8	15/76	19 · 7	0 · 52
Sought treatment in past 2 weeks	8/40	20 · 0	4/38	10 · 5	0 · 25	20/82	24 · 4	17/76	22 · 4	0 · 76
	8 & 10 weeks		8 & 10 weeks			12 & 14 weeks		12 & 14 weeks		
Cough	6/80	7 · 5	6/76	7 · 9	0 · 92	8/84	9 · 5	2/82	2 · 4	0 · 06
Diarrhoea	23/80	28 · 7	21/76	27 · 6	0 · 88	28/84	33 · 3	19/82	23 · 2	0 · 15
Refuse breast	1/80	1 · 2	3/76	3 · 9	0 · 28	0/84	0 · 0	1/82	91 · 2	0 · 31
Feverishness	19/80	23 · 7	26/76	34 · 2	0 · 15	14/84	16 · 7	8/82	9 · 8	0 · 19
Sought treatment in past 2 weeks	17/80	21.2	13/76	17.1	0.51	16/84	19.0	6/82	7.3	0.03
	16 & 18 weeks		16 & 18 weeks			20 & 22 weeks		20 & 22 weeks		
Cough	3/79	3 · 8	4/81	4 · 9	0 · 70	1/73	1 · 4	5/81	6 · 2	0 · 12
Diarrhoea	20/79	25 · 3	19/81	23 · 5	0 · 78	17/73	23 · 3	19/81	23 · 5	0 · 98
Refuse breast	2/79	2 · 5	2/81	2 · 5	0 · 98	1/73	1 · 4	5/81	6 · 2	0 · 12
Feverishness	15/79	18 · 9	16/81	19 · 7	0 · 90	12/73	16 · 4	23/81	28 · 4	0 · 08
Sought treatment in past 2 weeks	14/79	17 · 7	12/81	14 · 8	0 · 62	15/73	20 · 5	16/81	19 · 7	0 · 90

^a^% of subjects with side effects as a percentage of the total number of visits for the periods indicated

There were no reports of convulsions and bulging fontanelles

## Discussion

This study explored the use of stable ^13^C-tracer methodology to assess the VA body pool size in infants who were followed for a 5-months period of micronutrient home fortification with and without VA. Stable isotopes were used because they lacked the potential harmful effects of radioisotopes on human health making them ideal for studying a broad range of metabolic conditions [47]. The baseline VA status of the children who participated was normal as assessed by the MRDR test and the VA status remained normal after the intervention. The mean TBS of VA also did not differ between groups after the intervention. Serum retinol concentrations were <0.7 μmol/L in 34.9 % of the children at baseline, but this may have been due to the fact that ~23 % of them had inflammation, which was assessed with CRP.

The approach of using two different methods of assessing vitamin A status at different times meant that it was not possible to compare VA body pool size at any one point using the ^13^C-RID test. The only option was to compare the VA body pool size of children in the intervention and the control group. This may have limited our power to detect an intervention effect. For ethical reasons, our comparative group was not a true placebo but received other micronutrients like zinc and iron, which are also known to enhance VA status and could have led to the lack of an intervention effect. Zinc is a cofactor in the β-carotene cleavage enzyme potentially making the VA more bioavailable from plant sources [48]. Zinc also is involved in the synthesis of retinol-binding protein and thus may influence transport [49].

The VA status of young infants is known to be influenced by the liver retinol stores at birth, consumption of VA from breast milk and other foods, and losses from infections and parasites [50]. Many infants in developing countries remain VA deficient at 6 months of age after the weaning period and will require additional VA [51, 52]. The present study showed that infants in the two groups had comparable VA status as assessed by the MRDR at baseline and ^13^C-RID tests at endline. The fact that there was no difference in the vitamin A status of infants between groups suggests that in rural communities in Ghana, this method of home fortification did not significantly improve the VA stores of infants in the intervention group compared with infants in the control group over the study period. This is likely due to the adequate VA status observed in this study. TBS were twice as high as Thai children who had marginal to deficient liver reserves and had no access to fortified foods [40], and half as much as Zambian children who had adequate to hypervitaminotic stores of VA on the background of VA supplementation and fortification [41]. The mean values obtained in these Ghanaian infants (~0.8 μmol/g liver) are the same as the midpoint of two Ghanaian infants (0.77 μmol/g liver) who died from serious infections [53]. In 6 to 12 month old US children, the mean value of vitamin A 0.30 ± 0.21 μmol/g liver is lower than the assessed vitamin A values with Ghanaian infants [54]. Ghana had many VA interventions in place when this trial occurred including VA supplements at immunisation contacts and post-partum supplementation to lactating mothers, which may have been missed on the identity cards. Furthermore, green leafy vegetables are widely consumed [38] and vegetable oil and wheat flour are now VA-fortified [55].

Three main strategies have been implemented for improving the VA status among populations: supplementation, food fortification, and dietary diversification [56]. De Pee et al. emphasised the need for effective VA programs in poor countries to include a mix of supplementation, fortification, and dietary diversification [57]. Filteau and Tomkins have advocated that the choice of strategy is context specific and must take into account climate, the agricultural potential of the region, local infrastructure, food beliefs, and socioeconomic status of the population [56]. The current study reveals that sensitive VA assessment is also necessary to demonstrate whether supplementation or fortification is needed in target groups.

Young infants from developing countries are often VA deficient and studies from Bangladesh and Brazil have shown that a quarter to 90 % of children studied had inadequate liver stores assessed by MRDR and autopsy samples, respectively [58]. However, studies in American infants reported no VA deficiency in livers of 6–12 month old infants at necropsy [59]. Assessment of morbidity during our trial did not reveal any difference between those who had been given Sprinkles with VA and those whose sachets did not contain VA. This observation is similar to that found in a trial in Ghana where children were followed weekly to ascertain the occurrence of morbidity. There were no significant differences between the two arms (vitamin A and placebo) with respect to diarrheal and respiratory conditions, but children who received VA had significantly fewer clinical visits and hospital admissions [12]. Villamor and Fawzi have suggested that the protective effect of VA was mediated by a reduction in severity rather than the incidence of infections [60] and this study seems to agree with those findings even though in our study seeking hospital care was used as a proxy for severity. In a trial in Tanzania by Idindili et al., a clinical surveillance system did not confer any clinically important absolute effect on morbidity [61] and this was seen in earlier trials in Ghana in Kintampo [29] but in that trial there were differences in all anthropometric indices between the vitamin A and the placebo groups. The earlier study in Ghana enrolled younger children and gave three doses of 25,000 IU VA at 6, 10 and 14 weeks of age with immunization compared with children in this present study who were between the ages 7–9 months at enrolment. The impact of improvements in VA status is also likely to be related to the extent of deficiency in the population [12, 21]. It should be noted that mothers in the study communities had low socioeconomic status and results of the MRDR tests conducted at baseline showed that all infants had adequate VA status. The mothers may have received high dose supplements post-partum and this may have benefited the infants but no evidence exists to confirm whether this actually happened. No evidence existed, but the infants enrolled in the study may have received VA at their earlier immunization contacts. However, breast-feeding is universal and likely contributed to the adequate liver stores in these children.

Vitamin A can be obtained from the diet as preformed VA (retinol and its esterified form, retinyl ester) in dairy and organ meats or as provitamin A carotenoids from vegetable and fruits; although, it is unlikely that infants in the study consumed much of these foods. In developing countries, 70–90 % of VA is obtained from provitamin A carotenoids in plant foods and these are absorbed much less efficiently (20–50 %) depending on VA status and other non-dietary factors [48, 62]. Some of the main staple foods in the studied area in Ghana are millet, sorghum, and groundnuts, which do not contain significant amounts of carotenoids. However, in Europe and the United States, 75 % of dietary VA is from preformed VA and the fortification of foods, such as milk, breakfast cereals, and some snack foods [63]. This study was not able to do more sophisticated comparisons with the MRDR and stable isotope dilution, such as sensitivity and specificity, because there was a significant amount of time in between the MRDR and isotope dilution tests. Future comparisons of biomarkers should consider this. Developing countries, such as Ghana, have often used serum retinol in the assessment of VA status but stable isotope methodology can be used, even though it is more expensive [27], to quantitatively estimate TBS of VA [21, 43].

## Conclusions

This is the first study to assess VA status using the MRDR test before and stable isotope dilution technique after the intervention in the same children. Both the MRDR and ^3^C-RID tests confirmed that the infants had adequate VA status before and after home fortification of their complementary foods. These tests offered more information than serum retinol concentrations alone, which indicated VA deficiency. In fact, 34·9 % of the children were diagnosed with VA deficiency using serum retinol at baseline, which WHO defines as a severe public health problem. This is one of the reasons that WHO recommends that serum retinol concentrations should not be used alone as they are homeostatically controlled and do not change unless VA status is deficient [64]. Due to this phenomenon, other assays, such as the MRDR and RID tests, have been developed. In current studies using only serum retinol concentrations to assess VA status, it is highly recommended that CRP and α_1_-acid glycoprotein be measured to correct serum retinol concentrations [65]. Further population-based research needs to be conducted to determine the feasibility of using stable isotopes to evaluate different VA interventions [66].

## Abbreviations

^13^C-RID: ^13^C-retinol isotope dilution
CRP: C-reactive protein
DR: 3,4-didehydroretinol
GCCIRMS: gas chromatography-combustion-isotope ratio mass spectrometer
MRDR: modified relative dose response
R: retinol
TBS: total body stores
VA: Vitamin A
